# Exploring the Link between Cardiorenal and Metabolic Diseases

**DOI:** 10.3390/healthcare11212831

**Published:** 2023-10-26

**Authors:** Luis D’Marco, Ana Checa-Ros

**Affiliations:** Grupo de Enfermedades Cardiorrenales y Metabólicas, Departamento de Medicina y Cirugía, Universidad Cardenal Herrera-CEU, CEU Universities, Carrer Lluis Vives, 1, 46115 Valencia, Spain

## 1. Introduction

**Figure 1:**
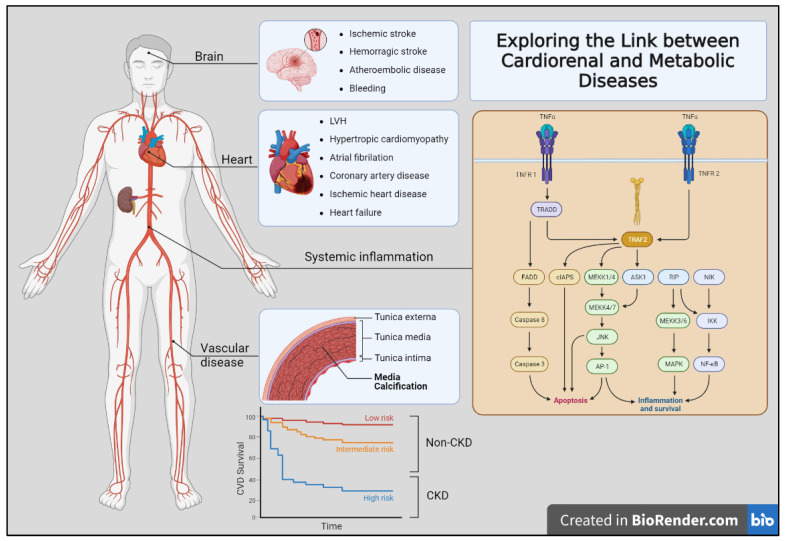
Exploring the link between cardiorenal and metabolic diseases. The figure shows the multiple clinical complications observed in organ-specific (brain, heart, and vascular bed) affectation. Additionally, several mechanisms contribute to the development of these diseases, including chronic inflammation, oxidative stress, and endothelial dysfunction. Moreover, the non-CKD population showed lower to intermediate risk (red and yellow lines) of cardiovascular disease risk than those patients with CKD (blue line).

The close link between metabolic diseases, such as obesity and diabetes mellitus, and cardiorenal disease can be attributed not only to direct risk factors, such as hypertension, but also to the intricate interplay of various pathophysiological processes. These metabolic conditions contribute to the development and progression of cardiorenal diseases through multiple mechanisms, including the activation of pro-inflammatory and pro-atherogenic pathways mediated by oxidative stress and inflammation [1,2] (Figure 1). When examining this relationship, chronic kidney disease (CKD) itself can foster a pro-inflammatory state characterized by increased oxidative stress and insulin resistance. As CKD progresses, it disrupts normal metabolic processes, ultimately leading to an elevated risk of cardiovascular events and mortality [3]. Thus, gaining a comprehensive understanding of the kidney–heart axis and its associated alterations becomes paramount in effectively managing and addressing the cardiorenal complications stemming from CKD. By delving deeper into the intricate connections between the kidney and heart, medical professionals can implement more accurate and proactive approaches to patient care.

Central to this pursuit is the identification of biomarkers capable of detecting preclinical stages of cardiorenal disease. Early detection can significantly improve patient outcomes, allowing for timely intervention and preventive measures to halt disease progression. By identifying specific biomarkers indicative of impending cardiorenal complications, healthcare providers can tailor treatments and strategies to the individual needs of each patient, thus enhancing the overall quality of care [4,5]. Additionally, a better comprehension of the kidney–heart axis opens doors to exploring novel therapeutic targets and interventions. Researchers can delve into innovative treatment modalities aimed at mitigating the impact of metabolic conditions on cardiorenal health.

An interdisciplinary approach involving nephrologists, cardiologists, endocrinologists, and researchers will facilitate the development of more effective and personalized treatment regimens. Accordingly, collaboration among medical professionals, academic institutions, and research centers becomes pivotal. Data sharing and joint efforts in conducting clinical studies will strengthen our understanding of these complex relationships. Moreover, fostering dialogue between various stakeholders will promote knowledge exchange, leading to innovative breakthroughs in the field [6]. Thus, in this Special Issue called “Exploring the Link between Cardiorenal and Metabolic Diseases”, we want to highlight some important aspects of these complex interactions.

## 2. Current State of Knowledge of Relationship between Metabolic and Cardiorenal Diseases

The relationship between metabolic conditions and cardiorenal diseases has been fairly well studied. Thus, metabolic conditions, such as obesity, type 2 diabetes, and dyslipidemia, have been recognized as significant risk factors for the development and progression of cardiorenal diseases; moreover, these link is multifactorial. As we explained earlier, several mechanisms contribute to the development of these diseases, including chronic inflammation, oxidative stress, endothelial dysfunction, and renin–angiotensin–aldosterone system (RAAS) hyper-activation. Additionally, shared risk factors, such as a sedentary lifestyle, unhealthy diet, and genetic predisposition, further contribute to the interplay between these conditions [7,8].

In this sense, CKD patients’ manifestations of cardiorenal disease can be broadly divided into those affecting primary organs such as the heart, kidneys, and brain, and those affecting the blood vessels. Moreover, these processes are not mutually exclusive. Traditional risk factors for atherosclerotic are insufficient to explain this vastly increased risk. Therefore, the contribution of CKD-specific risk factors has been postulated including anemia, abnormal bone mineral metabolism, oxidative stress, and chronic inflammation. Finally, deep knowledge about the impact of the cardiovascular risk burden in CKD-affected patients may help to improve their higher rates of mortality [9] (Figure 1).

Currently, to manage and prevent cardiorenal diseases in individuals with metabolic alterations, a comprehensive approach is crucial. This approach involves lifestyle modifications (e.g., regular physical activity, healthy diet), control of blood pressure and blood sugar levels, appropriate medication use, and regular medical check-ups to monitor kidney and heart health [10].

## 3. The Existing Gaps and Challenges in Metabolic, Cardiac, and Renal Diseases

These gaps and challenges highlight areas where further research and attention are needed to improve our understanding and management of these conditions. Some of the key gaps and challenges include:

- ***Early Detection and Prevention:*** Early detection of metabolic conditions and their impact on the cardiovascular and renal systems is crucial for effective prevention. There is a need for better biomarkers and diagnostic tools to identify individuals at risk of developing cardiorenal diseases due to metabolic conditions [10].
- ***Causal Relationships and Mechanisms:*** While metabolic conditions and cardiorenal diseases are interconnected, establishing causal relationships and understanding the underlying mechanisms remain challenging. The exact pathways through which metabolic disorders contribute to the development of cardiorenal diseases require further investigation [11].
- ***Risk Prediction Models:*** Developing accurate risk prediction models that incorporate various metabolic and clinical factors is an ongoing challenge. Such models can help identify high-risk individuals and facilitate targeted interventions to prevent or delay the onset of cardiorenal diseases.
- ***Patient Heterogeneity:*** Patients with metabolic conditions and cardiorenal diseases exhibit significant heterogeneity in their response to treatment and disease progression. Understanding this variability is essential for personalized and effective patient care.
- ***Management Strategies:*** While lifestyle modifications and pharmacological interventions play a crucial role in managing metabolic conditions and their impact on the heart and kidneys, optimizing treatment strategies for individual patients remains a challenge [11].
- ***Long-term Outcomes:*** There is a need for more long-term studies to assess the impact of metabolic conditions on cardiorenal health over extended periods. This information can help identify potential interventions that might prevent or slow down disease progression.
- ***Translational Research:*** Bridging the gap between basic science research and clinical practice is essential for translating scientific findings into effective therapies and interventions for patients with metabolic-related cardiorenal diseases [11].
- ***Comorbidities and Polypharmacy:*** Patients with metabolic conditions and cardiorenal diseases often have multiple comorbidities, leading to complex treatment regimens and potential drug interactions. Coordinating care for these individuals can be challenging [6,12].
- ***Access to Care and Health Disparities:*** Disparities in healthcare access and outcomes based on socioeconomic status, race, and geographical location can influence the management and outcomes of metabolic conditions and cardiorenal diseases.
- ***Data Sharing and Collaboration:*** Sharing data and fostering collaboration among researchers and healthcare providers are critical for advancing our understanding of these conditions and developing comprehensive treatment strategies [13].

Finally, it is essential to address these gaps and challenges to improve the prevention, diagnosis, and management of cardiorenal diseases in individuals with metabolic conditions. Ongoing research, technological advancements, and a multidisciplinary approach involving clinicians, researchers, and policymakers will be instrumental in making progress in this field.

This Special Issue features papers on a broad range of topics as described below.

## 4. The Renal–Heart Axis: Arrhythmias

Atrial fibrillation (AF) is the most common arrhythmia in patients with CKD [14]. Its prevalence increases in relation to the decrease in kidney function and in the presence of albuminuria [15]. Cardio-embolic brain complications derived from AF, such as stroke, dementia, and Parkinson’s disease, would complicate the course and prognosis of CKD-affected patients, particularly those in advanced stages of the disease [16].

Vitamin K antagonist coagulants (VKAs) and direct-acting oral anticoagulants (DOACs) are the pharmacological options for the treatment of AF. However, there is a concern about the appearance of vascular calcifications and glomerular hematuria with the use of VKAs. At the same time, the pharmacokinetics and pharmacodynamics of most anticoagulant drugs represent a real challenge in the advanced stages of CKD, due to kidney failure and multiple drug–drug interactions [17].

The FAERC study was a retrospective, multicenter, and cohort study performed on patients with CKD (all stages) from 15 different hospitals in Spain that evaluate the prevalence of AF and the clinical implications of anticoagulant treatments. Thus, the data from 4749 CKD patients were analyzed, revealing an AF prevalence of around 12%. Of note, more than 98% of the patients were receiving anticoagulant treatment, mainly VKAs. Over the 60-month follow-up, around 6% of the sample experienced an ischemic or embolic event. The use of DOACs was associated with fewer cerebrovascular events, although no association was found between the type of anticoagulant and the risk of bleeding, coronary artery disease, or death. This study emphasizes the importance of the nephrologist’s involvement in the anticoagulation management of CKD patients with AF [18].

## 5. Cardiorenal Disease in Metabolic Disorders

Fabry disease (FD, OMIM 301500) is a rare, X-linked, lysosomal storage disorder caused by the absence or deficiency of the α-galactosidase A enzyme [19], resulting in the accumulation of glycolipids in different organs, such as the kidney (renal failure) and heart (hypertrophic cardiomyopathy) [20,21]. Enzyme replacement therapy (ERT) was the only therapeutic option for patients with FD for several years; however, the novel pharmacological chaperone Galafold^®^ (migalastat) could overcome some of the limitations encountered with ERT. The review conducted by Perretta et al. [22] reports the benefits in terms of a significant reduction in the left ventricular mass index and stabilization of renal function when switching from ERT to Migalastat^®^ in patients with FD, considering that Migalastat^®^ is recommended when the estimated glomerular filtration rate (eGFR) is >30 mL/min/1.73 m^2^.

## 6. Renal Damage in Non-Kidney Solid Organ Transplants

Kidney failure is a common complication in non-kidney solid organ transplant (NKSOT) patients, with an incidence that ranges between 7 and 21%, due to the development of histopathological changes, such as arterial hyalinosis and primary glomerular disease, and the adverse effects from the immunosuppression used to avoid organ rejection. The retrospective observational study performed by Viejo-Boyano et al. [23] aimed to identify the risk factors for increased serum creatinine ≥50%, renal replacement therapy (RRT), and death in the pre-transplant, peri-transplant, and post-transplant periods on a sample of 74 patients undergoing heart, liver, or lung transplants. Not being followed up by a nephrologist in the pre-transplant or peri-transplant periods was associated with a higher risk, measured as the worsening of kidney function (creatinine increase ≥50%). The risk to progress to end-stage CKD varied depending on the transplanted organ, with a lung transplant conferring a higher risk than a liver or heart transplant. Peri-transplant mechanical ventilation, peri-transplant and post-transplant anticalcineurin overdose, nephrotoxicity, and the number of hospital admissions were also significantly associated with a creatinine increase of ≥50% and developing end-stage CKD. These data serve to emphasize the need for an early and close follow-up by a nephrologist in patients receiving an NKSOT.

## 7. Anti-Inflammatory Synergy for the Metabolic Complications from Chronic Kidney Disease (CKD)

Secondary hyperparathyroidism (SHP) is a frequent and important complication of patients suffering from CKD, particularly in those receiving renal replacement treatments [24,25]. Elevated serum parathyroid hormone (PTH) contributes to bone and cardiovascular disorders and worsens the pro-inflammatory state of patients with CKD [26]. Through an observational study on 142 patients receiving hemodialysis, D’Marco et al. [27] compared the effects on PTH levels and inflammatory markers between three therapeutic groups: (1) the novel calcimimetic agent etelcalcetide; (2) the vitamin D analog paricalcitol; and (3) the combination of both treatments. The combined treatment (etelcalcetide + paricalcitol) proved to safely reduce PTH levels, with a significant power to reduce primary inflammatory markers, such as the C-reactive protein (CRP). These results argue for further clinical trials exploring the benefits of this combination to alleviate the pro-inflammatory status of patients with CKD and, consequently, the cardiovascular risk in this population of patients.

## 8. Conclusions

In conclusion, recognizing the intricate connections between metabolic diseases, cardiorenal health, and associated complications unveils a realm of possibilities for enhancing patient care. Through a multidisciplinary approach, focused research, and a commitment to improving early detection, we can stride towards better outcomes for those impacted by these conditions. Embracing the evolving landscape of the kidney–heart axis holds the key to unlocking advancements that will redefine the future of cardiorenal disease management.

## References

[1] Andreadi A., Bellia A., Daniele N.D., Meloni M., Lauro R., Della-Morte D., Lauro D. (2022). The molecular link between oxidative stress, insulin resistance, and type 2 diabetes: A target for new therapies against cardiovascular diseases. Curr. Opin. Pharmacol..

[2] Zhang R., Mamza J.B., Morris T., Godfrey G., Asselbergs F.W., Denaxas S., Hemingway H., Banerjee A. (2022). Lifetime risk of cardiovascular-renal disease in type 2 diabetes: A population-based study in 473,399 individuals. BMC Med..

[3] Tomás-Simó P., D’Marco L., Romero-Parra M., Tormos-Muñoz M.C., Sáez G., Torregrosa I., Estañ-Capell N., Miguel A., Gorriz J.L., Puchades M.J. (2021). Oxidative Stress in Non-Dialysis-Dependent Chronic Kidney Disease Patients. Int. J. Environ. Res. Public Health.

[4] Chen C., Yang X., Lei Y., Zha Y., Liu H., Ma C., Tian J., Chen P., Yang T., Hou F.F. (2016). Urinary Biomarkers at the Time of AKI Diagnosis as Predictors of Progression of AKI among Patients with Acute Cardiorenal Syndrome. Clin. J. Am. Soc. Nephrol..

[5] Chung E.Y.M., Trinh K., Li J., Hahn S.H., Endre Z.H., Rogers N.M., Alexander S.I. (2022). Biomarkers in Cardiorenal Syndrome and Potential Insights Into Novel Therapeutics. Front. Cardiovasc. Med..

[6] Jha V., Arici M., Collins A.J., Garcia-Garcia G., Hemmelgarn B.R., Jafar T.H., Pecoits-Filho R., Sola L., Swanepoel C.R., Tchokhonelidze I. (2016). Understanding kidney care needs and implementation strategies in low- and middle-income countries: Conclusions from a “Kidney Disease: Improving Global Outcomes” (KDIGO) Controversies Conference. Kidney Int..

[7] Tikkanen I., Narko K., Zeller C., Green A., Salsali A., Broedl U.C., Woerle H.J. (2015). EMPA-REG BP Investigators. Empagliflozin reduces blood pressure in patients with type 2 diabetes and hypertension. Diabetes Care.

[8] Forman D.E., Butler J., Wang Y., Abraham W.T., O’Connor C.M., Gottlieb S.S., Loh E., Massie B.M., Rich M.W., Stevenson L.W. (2004). Incidence, predictors at admission, and impact of worsening renal function among patients hospitalized with heart failure. J. Am. Coll. Cardiol..

[9] Bover J., Aguilar A., Arana C., Molina P., Lloret M.J., Ochoa J., Berná G., Gutiérrez-Maza Y.G., Rodrigues N., D’Marco L. (2021). Clinical Approach to Vascular Calcification in Patients with Non-dialysis Dependent Chronic Kidney Disease: Mineral-Bone Disorder-Related Aspects. Front. Med..

[10] Handelsman Y., Butler J., Bakris G.L., DeFronzo R.A., Fonarow G.C., Green J.B., Grunberger G., Januzzi J.L., Klein S., Kushner P.R. (2023). Early intervention and intensive management of patients with diabetes, cardiorenal, and metabolic diseases. J. Diabetes Complicat..

[11] Bello A.K., Alrukhaimi M., Ashuntantang G.E., Basnet S., Rotter R.C., Douthat W.G., Kazancioglu R., Köttgen A., Nangaku M., Powe N.R. (2017). Complications of chronic kidney disease: Current state, knowledge gaps, and strategy for action. Kidney Int. Suppl..

[12] Nichols G.A., Romo-LeTourneau V., Vupputuri S., Thomas S.M. (2019). Delays in anti-hyperglycaemic therapy initiation and intensification are associated with cardiovascular events, hospitalizations for heart failure and all-cause mortality. Diabetes Obes. Metab..

[13] Levin A., Tonelli M., Bonventre J., Coresh J., Donner J.A., Fogo A.B., Fox C.S., Gansevoort R.T., Heerspink H.J.L., Jardine M. (2017). Global kidney health 2017 and beyond: A roadmap for closing gaps in care, research, and policy. Lancet.

[14] Soliman E.Z., Prineas R.J., Go A.S., Xie D., Lash J.P., Rahman M., Ojo A., Teal V.L., Jensvold N.G., Robinson N.L. (2010). Chronic Kidney Disease and Prevalent Atrial Fibrillation: The Chronic Renal Insufficiency Cohort (CRIC). Am. Heart J..

[15] Alonso A., Lopez F.L., Matsushita K., Loehr L.R., Agarwal S.K., Chen L.Y., Soliman E.Z., Astor B.C., Coresh J. (2011). Chronic Kidney Disease Is Associated with the Incidence of Atrial Fibrillation. Circulation.

[16] Rosca C.I., Sharma A., Nisulescu D.-D., Otiman G., Duda-Seiman D.-M., Morariu S.I., Lighezan D.F., Kundnani N.R. (2023). Prevalence of Cardio-Embolic Brain Complications in Permanent and Paroxysmal Atrial Fibrillation Patients. Healthcare.

[17] Jain N., Reilly R.F. (2018). Clinical Pharmacology of Oral Anticoagulants in Patients with Kidney Disease. Clin. J. Am. Soc. Nephrol..

[18] Montomoli M., Roca L., Rivera M., Fernandez-Prado R., Redondo B., Camacho R., Moyano C., Pampa S., Gonzalez A., Casas D. (2022). Oral Anticoagulation in Patients with Chronic Kidney Disease and Non-Valvular Atrial Fibrillation: The FAERC Study. Healthcare.

[19] Germain D.P. (2010). Fabry disease. Orphanet J. Rare Dis..

[20] Ortiz A., Germain D.P., Desnick R.J., Politei J., Mauer M., Burlina A., Eng C., Hopkin R.J., Laney D., Linhart A. (2018). Fabry disease revisited: Management and treatment recommendations for adult patients. Mol. Genet. Metab..

[21] Arends M., Wanner C., Hughes D., Mehta A., Oder D., Watkinson O.T., Elliott P.M., Linthorst G.E., Wijburg F.A., Biegstraaten M. (2017). Characterization of Classical and Nonclassical Fabry Disease: A Multicenter Study. J. Am. Soc. Nephrol..

[22] Perretta F., Jaurretche S. (2023). Fabry Disease: Switch from Enzyme Replacement Therapy to Oral Chaperone Migalastat: What Do We Know Today?. Healthcare.

[23] Viejo-Boyano I., López-Romero L.C., D’Marco L., Checa-Ros A., Peris-Fernández M., Garrigós-Almerich E., Ramos-Tomás M.C., Peris-Domingo A., Hernández-Jaras J. (2023). Role of the Nephrologist in Non-Kidney Solid Organ Transplant (NKSOT). Healthcare.

[24] Torres P.A.U., De Broe M. (2012). Calcium-sensing receptor, Calcimimetics, and Cardiovascular Calcifications in Chronic Kidney Disease. Kidney Int..

[25] Levin A., Bakris G.L., Molitch M., Smulders M., Tian J., Williams L.A., Andress D.L. (2007). Prevalence of Abnormal Serum Vitamin D, PTH, Calcium, and Phosphorus in Patients with Chronic Kidney Disease: Results of the Study to Evaluate Early Kidney Disease. Kidney Int..

[26] Herzog C.A., Asinger R.W., Berger A.K., Charytan D.M., Díez J., Hart R.G., Eckardt K.-U., Kasiske B.L., McCullough P.A., Passman R.S. (2011). Cardiovascular Disease in Chronic Kidney Disease. A Clinical Update from Kidney Disease: Improving Global Outcomes (KDIGO). Kidney Int..

[27] D’Marco L., Checa-Ros A., Gamero D., Soto C., Salazar J., Nava M., Bermúdez V., Dapena F. (2023). Etelcalcetide and Paricalcitol in Chronic Kidney Disease: When the Target Is Inflammation. Healthcare.

